# Metabolomic Profiling of Blood Plasma in Females with Hyperplasia and Endometrial Cancer

**DOI:** 10.3390/metabo14020109

**Published:** 2024-02-06

**Authors:** Hicham Benabdelkamel, Malak A. Jaber, Khalid Akkour, Reem H. AlMalki, Assim A. Alfadda, Afshan Masood, Salini Scaria Joy, Hani Alhalal, Moudi A. Alwehaibi, Maria Arafah, Eman Alshehri, Anas M. Abdel Rahman

**Affiliations:** 1 Proteomics Resource Unit, Obesity Research Center, College of Medicine, King Saud University, Riyadh 11461, Saudi Arabia; aalfadda@ksu.edu.sa (A.A.A.); afsmasood@ksu.edu.sa (A.M.); 441203139@student.ksu.edu.sa (M.A.A.); 2 Pharmaceutical Medicinal Chemistry & Pharmacognosy, Faculty of Pharmacy and Medical Sciences, University of Petra, Amman 1196, Jordan; malak.jaber@uop.edu.jo; 3 Obstetrics and Gynecology Department, College of Medicine, King Saud University Medical City, King Saud University, Riyadh 11461, Saudi Arabia; kakkour@ksu.edu.sa (K.A.); halhalal@ksu.edu.sa (H.A.); emhalshehri@ksu.edu.sa (E.A.); 4 Department of Botany and Microbiology, College of Science, King Saud University, Riyadh 11461, Saudi Arabia; rgalmalki@kfshrc.edu.s; 5 Department of Medicine, College of Medicine, King Saud University Medical City, King Saud University, Riyadh 11461, Saudi Arabia; 6 Department of Pathology, College of Medicine, King Saud University Medical City, King Saud University, Riyadh 11461, Saudi Arabia; mariaarafah@ksu.edu.sa; 7 Metabolomics Section, Department of Clinical Genomics, Center for Genome Medicine, King Faisal Specialist Hospital and Research Centre (KFSHRC), Riyadh 11211, Saudi Arabia

**Keywords:** endometrial cancer, hyperplasia (HP), metabolomics, LC-HRMS, energy metabolism

## Abstract

Uterine cancer is the most prevalent gynecologic malignancy in women worldwide. Endometrial cancer (EC) has an 81% five-year survival rate, depending on disease stage and time of diagnosis. While endometrial cancer is largely treatable when detected early, no established screening techniques are available in clinical practice. As a result, one of the most significant issues in the medical field is the development of novel ways for early cancer identification, which could boost treatment success rates. Liquid chromatography–high-resolution mass spectrometry (LC-HRMS)-based metabolomics was employed to explore the metabolomic markers and pathways unique to this cancer type and link them to the benign endometrial hyperplasia that may progress to cancer in 5% to 25% of patients. The study involved 59 postmenopausal participants, 20 with EC type 1, 20 with benign hyperplasia, and 19 healthy participants. Metabolite distribution changes were analyzed, and 338 of these features were dysregulated and significant. The first two main components, PC1 and PC2, were responsible for 11.5% and 12.2% of the total metabolites, respectively. Compared with the control group (CO), EC samples had 203 differentially expressed metabolites (180 upregulated and 23 downregulated); in hyperplasia (HP), 157 metabolites were dysregulated (127 upregulated and 30 downregulated) compared to the CO group while 21 metabolites exhibited differential regulation (16 upregulated and 5 downregulated) in EC plasma samples compared to the HP group. Hyperplasia samples exhibited similar metabolic changes to those reported in cancer, except for alterations in triglyceride levels, 7a,12 b-dihydroxy-5b-Cholan-24-oic acid, and Hept-2-enedioyl carnitine levels. The metabolites N-heptanoyl glycine and -(Methylthio)-2,3-isopentyl phosphate and formimino glutamic acid can be specific markers for hyperplasia conditions and dimethyl phosphatidyl ethanolamine and 8-isoprostaglandin E2 can be specific markers for EC conditions. Metabolic activities rely on mitochondrial oxidative phosphorylation for energy generation. The changes in metabolites identified in our study indicate that endometrial cancer cells adopt alternative strategies to increase energy production to meet the energy demand, thereby supporting proliferation.

## 1. Introduction

Endometrial cancer (EC) is commonly referred to as uterine cancer. It is the fifteenth most common cancer worldwide and the sixth most prevalent cancer in women, according to the World Cancer Research Fund International in 2020 [1]. EC is a disease in which malignant cells form in the endometrium’s tissues and the uterus lining, and it is considered the most common gynecologic cancer in females [2]. Uterine cancer diagnoses are increasing by over 2% annually in women under the age of 49 and by 1% in women over the age of 49 in the United States [3]. There are two primary subtypes of endometrial tumor based on clinical and endocrine features, I and II. Type I tumors represent the most frequent subtype, typically include estrogen-related characteristics, are low grade, and have a fair prognosis. Endometrial hyperplasia usually precedes the lesions, which are generally well-differentiated. Type II tumors, in contrast, represent less than 15% of ECs, a heterogeneous, poorly differentiated collection of high-grade festering tumors [4,5,6].

Based on histopathological characteristics, they are classified as endometrioid, serous, or clear-cell adenocarcinoma and into different grades depending on how much tumor cells resemble normal cells. Grade 1 (low-grade) cells resemble normal cells, exhibiting slow growth and limited spread. Grade 2 (moderate-grade) cells appear more aberrant and have higher metastatic potential. Grade 3 (high-grade) cells display extreme abnormalities, rapid growth, and increased metastasis [5]. The American Cancer Society (ACS) estimates that the 5-year relative survival rate for early-stage EC is 84% [7]. However, the clinical grade determination is challenging due to histologic subtype overlap. A more detailed categorization integrating molecular criteria has been introduced to enhance clinical decisions, optimize treatment plans, and elevate survival rates [8,9]. Irregular vaginal bleeding in women before menopause and regular vaginal bleeding through menopause and pain in the pelvic area, especially during urination or sexual intercourse, are signs and symptoms of EC. Early identification increases the likelihood that a malignancy will be successfully treated [10,11].

Metabolomics has emerged as a transformative force in biomarker discovery, significantly mitigating the morbidity and mortality associated with a broad spectrum of disorders and diseases. Its impact, notably in the context of cancer, as elucidated by the comprehensive study conducted by Jacob et al., cannot be overstated [12]. This study delves into the critical endeavor of identifying distinct metabolic markers and pathways inherently tied to various cancer types and their stages, representing a linchpin in contemporary cancer research. Within this context, cancer metabolomics holds vast potential, spanning risk assessment, early detection, the refinement of disease staging, the implementation of personalized prognostic and diagnostic modalities, and the vigilance in monitoring the efficacy of therapeutic interventions, all while considering the emergence of potential resistance mechanisms [13,14]. Strong evidence that cancer develops in the context of severe metabolic dysfunction is provided by significant risk factors for EC, including obesity, diabetes mellitus, hypertension, and estrogen exposure. For example, in large cohort research, a metabolomic approach was studied and validated for the screening of endometrial cancer using serum samples acquired from women scheduled for gynecological surgery. The study findings revealed that EC patients’ serum metabolomes were characterized by reduced levels of serine, glutamic acid, phenylalanine, and glyceraldehyde 3-phosphate, suggesting that these metabolites could be utilized as a low-cost, non-invasive, and accurate preliminary screening test for EC [15]. Another study discovered that sphingomyelin and glycine were statistically linked to an increased risk of endometrial cancer [16]. In another study, researchers were able to discriminate between recurrent and non-recurrent cases following surgery in postmenopausal women by combining 2-oleoylglycerol and TAG 42:2-FA12:0. This could be used to define clinically relevant risk categories following surgery [17]. Therefore, metabolomics research on EC is expected to provide insightful data regarding the condition [12,18,19].

The investigation at hand hinged upon using a label-free liquid chromatography–mass spectrometry (LC-MS)-based untargeted metabolomics approach. The principal objective was to discern whether a discernible aberration existed in the serum metabolite profiles among preoperative endometrial cancer (EC) cases when juxtaposed with patients diagnosed with uterine hyperplasia and individuals of sound health. This pursuit holds the promise of shedding light on distinctive metabolic signatures that serve as diagnostically and prognostically pertinent indicators in EC management and, potentially, in the broader context of oncology.

## 2. Materials and Methods

### 2.1. Population and Study Design

The King Saud University College of Medicine’s institutional review board examined and approved the study’s methods and procedures (IRB number: E-193622). The study’s participants provided their written, informed consent. This study was conducted from May 2012 to November 2022 at King Khalid University Hospital (KKUH) in Saudi Arabia. This study comprised patients with EC (n = 20, mean age is 62 ± 9), hyperplasia (n = 20, mean age is 61 ± 5), and healthy controls (n = 19, mean age is 59 ± 4) (Supplementary Table data S1). EC and hyperplasia diagnoses were confirmed based on histological examination. At the same time, the control participants were healthy women who were receiving a routine checkup at a gynecological clinic. Women who were fertile and those with a history of a prior cancer diagnosis in any other place were excluded from the study. Participants’ blood samples were drawn using EDTA-coated tubes and centrifuged, and the produced plasma samples were stored at −80 °C until analysis.

### 2.2. Metabolite Extraction

Metabolites were extracted using the protein precipitation reported previously [20,21]. In brief, 100 µL of plasma was mixed with 900 µL of 50% extraction solvent (acetonitrile: methanol). The samples were vortexed in a Thermomixer (Eppendorf, Hamburg, Germany) at 600 rpm, 4 °C for one hour. Then, samples were centrifuged at 16,000 rpm for 10 min at 4 °C (Eppendorf, SE, Germany), and supernatants were placed into Eppendorf tubes and evaporated using a vacuum evaporator (SpeedVac; Christ, Germany). To re-suspend the dried samples, 100 µL of a 1:1 ratio of mobile phase A: B (A: 0.1% formic acid in dH_2_O and B: 0.1% formic acid in 50% MeOH and ACN) was used.

### 2.3. Metabolite Analysis

Metabolomics profiling was performed using untargeted metabolomics analyses by LC-HRMS, as previously reported [22]. Metabolites were acquired by a Waters ACQUITY ultrahigh-pressure liquid chromatography (UPLC) system coupled with a Xevo G2-S QTOF mass spectrometer equipped with an electrospray ionization source (ESI) in positive and negative modes (ESI+, ESI−). The metabolites were chromatographed using an ACQUITY UPLC XSelect (100 × 2.1 mm 2.5 μm) column (Waters Ltd., Elstree, UK). The mobile phases A and B were pumped to the column in a gradient mode (0–16 min 95–5% A, 16–19 min 5% A, 19–20 min 5–95% A, 20–22 min 5–95% A) at a 300 μL/min flow rate. MS conditions were as follows: the source temperature was 150 °C, the desolvation temperature was 500 °C (ESI+) or 140 °C (ESI−), capillary voltages were 3.20 kV (ESI+) or 3 kV (ESI−), cone voltage was 40 V, desolvation gas flow was 800.0 L/h, and cone gas flow was 50 L/h. The collision energy of low and high functions was set off at 10–50 V, respectively, in MS^E^ mode. The mass spectrometer was calibrated, as recommended by the vendor, with sodium formate in the range of 100–1200 Da in both ionization modes. Data-independent acquisition (DIA) was carried out in continuum mode with a Masslynx™ V4.1 workstation (Waters Inc., Milford, MA, USA).

### 2.4. Data Handling and Processing

The raw MS data were processed following a standard pipeline starting from alignment based on the *m*/*z* value and the ion signals’ retention time, peak picking, and signal filtering based on the peak quality using the Progenesis QI v.3.0 software from Waters (Waters Technologies, Milford, MA, USA).

Multivariate statistical analysis was evaluated using MetaboAnalyst v. 5.0 (McGill University, Montreal, QC, Canada) (http://www.metaboanalyst.ca, accessed on 30 July 2023) [23]. The imported datasets were normalized by median, Pareto-scaled, and log-transformed to maintain their normal distribution and then used to generate partial least squares discriminant analysis (PLS-DA) and orthogonal partial least squares discriminant analysis (OPLS-DA) models. The OPLS-DA models created were evaluated using the fitness of the model (R2Y) and predictive ability (Q2) values [23]. Univariate analysis was performed using Mass Profiler Professional software (Agilent, Santa Clara, CA, USA). One-way ANOVA (Tukey’s post hoc test, no correction *p* ≤ 0.05) was performed among groups. Venn diagrams were developed using MPP software (Agilent Inc., Santa Clara, CA, USA), and heatmap analysis for altered features was performed using Pearson’s distance measure.

The significant features obtained were annotated using the Human Metabolome Database (HMDB) based on the accurate precursor mass, the fragmentation pattern, and the isotopic distribution [24]. Exogenous compounds, such as drugs and food additives, were eliminated manually from the final list.

### 2.5. Bioinformatic Analysis

The functions of the identified metabolites differently expressed in EC vs. control and HP vs. control samples, as well as their interactions, were examined using Ingenuity Pathway Analysis (IPA). The software maps the IDs into the manually curated Intelligence Knowledge Base, containing information from all published scientific publications. By comparing the experimental expression of the data to known biological networks, this software identifies the activities and pathways that are substantially associated with the metabolite list.

## 3. Results

### 3.1. Mass Ion Detection and Metabolite Identification

A total of 9882 mass ion features were detected, with 6892 positive and 2990 negative ionization modes. After several filtration processes such as alignment, peak picking, missing value removal, and applying a filter by the frequency with a cutoff percentage of 80 of all samples, 6722 features remained. The 6722 ions were evaluated statistically, revealing that 338 metabolites were significantly dysregulated among all three groups. To confirm that all depicted data have a Gaussian distribution, the data were normalized by the median, log-transformed, and Pareto-scaled to eliminate systemic variances. Out of 338, only 102 metabolites were annotated using the Human Metabolome Database (HMDB). The exogenous metabolites (i.e., drugs, drug metabolites, environmental exposures) were excluded, and 33 endogenous metabolites were identified in all three groups. Out of 33 endogenous metabolites, 7 metabolites were common among both EC and HP (Supplementary data S2).

### 3.2. Overview of the Three Study Groups (Ctrl, HP, and EC)

**Figure 1:**
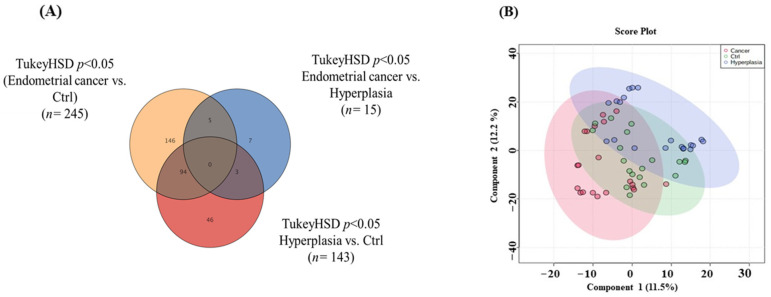
Comparison of identified metabolite features between the groups. (**A**) Venn diagram showing significant differences using one-way ANOVA (Tukey’s Post-hoc, no correction *p* < 0.05) of 3 groups (Endometrial cancer vs. Hyperplasia, Endometrial cancer vs. Ctrl, and Hyperplasia vs. Ctrl). (**B**) Partial least squares discriminant analysis (PLS-DA) displays semi-separation between groups. n is the number of metabolite features identified with a significant *p*-value (*p* < 0.05).

A Venn diagram was used to obtain an overview of the significantly altered ions between the groups. Venn diagram revealed that 338 metabolites were dysregulated in the three datasets (Figure 1A). Additionally, the PLS-DA model was generated to examine any sample clustering and group separation in the datasets and identify any possible outliers (Figure 1B).

### 3.3. Metabolomic Profiling between EC and Ctrl

**Figure 2:**
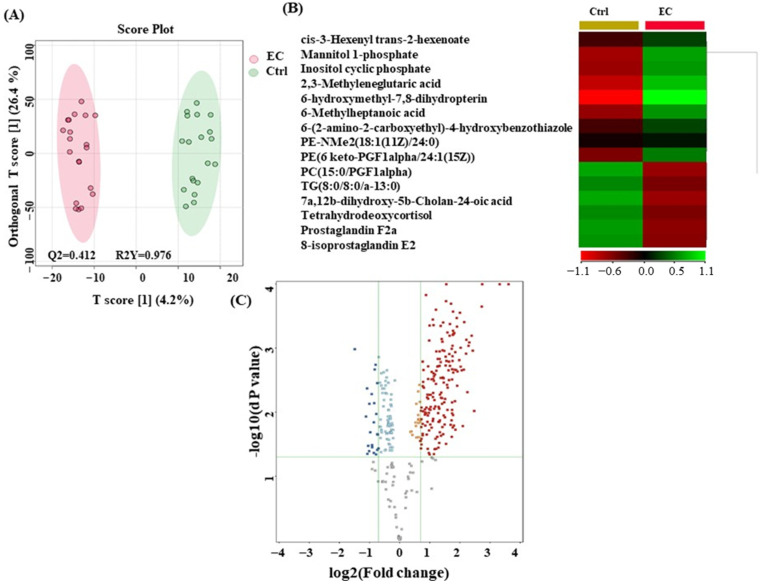
Metabolite profiling between endometrial cancer (EC) and control (Ctrl) patients. (**A**) Orthogonal partial least squares discriminant analysis (OPLS-DA) score plot showing evident separation between two groups (EC and Ctrl). The robustness of the created models was evaluated by the fitness of the model (R2Y = 0.976) and predictive ability (Q2 = 0.412) values. The EC and Ctrl samples are represented as red and green circles, respectively. (**B**) Heat map analysis of identified metabolites that were significantly altered between control (yellow) and EC (red) groups. The color range bar indicates downregulated metabolites as red and upregulated metabolites as green between EC and Ctrl groups. (**C**) The volcano plot shows a significant change in the levels of several metabolites. Red represents upregulated and blue describes downregulated plasma metabolites in EC compared with the control group (FDR *p*-value ≤ 0.05, fold change ≥ 1.5). The grey dots represent unsignificant metabolites.

The metabolites that distinguished between EC and Ctrl are displayed in Figure 2. OPLS-DA, a supervised multivariate approach, is shown in Figure 2A. The distinct separation of the EC group from Ctrl suggests that plasma metabolites may be useful for identifying EC. According to the heat map analysis, Figure 2B shows the metabolites that were significantly different between the Ctrl (yellow) and EC (red) groups. The metabolites with a substantial difference between the EC and Ctrl groups are represented on the heat map. As a result, they might be considered prospective metabolite biomarkers for EC detection. A moderate *t*-test (*p*-value < 0.05) and fold change (FC cutoff of 1.5) were used to analyze the volcano plot between the EC and Ctrl groups. The results showed that from 203 dysregulated metabolites, 180 (red) and 23 (blue) metabolites were upregulated and downregulated in the EC and Ctrl groups, respectively. A total of 15 metabolites were identified as human endogenous metabolites.

### 3.4. Metabolomics Profiling between HP and Ctrl

**Figure 3:**
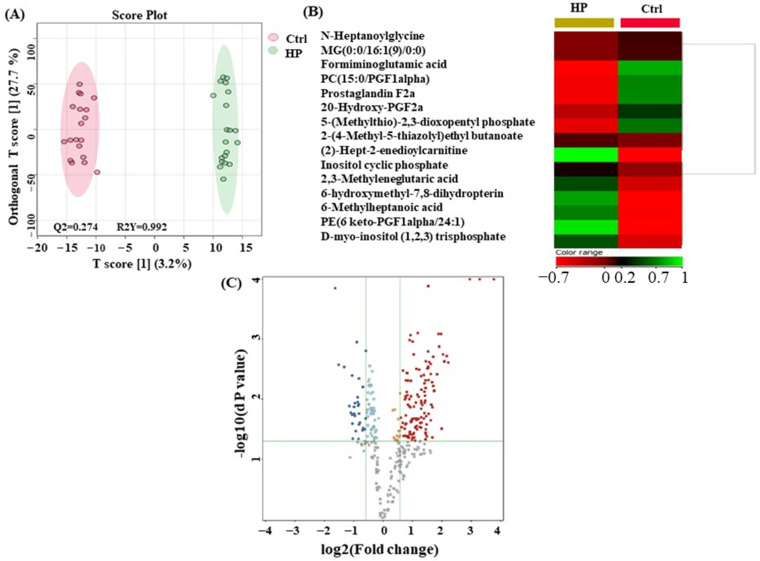
Metabolomics profiling between hyperplasia (HP) and control (Ctrl) groups. (**A**) Orthogonal partial least squares discriminant analysis (OPLS-DA) score plot showing evident separation between HP and Ctrl groups. The robustness of the created models was evaluated by the fitness of the model (R2Y = 0.992) and predictive ability (Q2 = 0.274) values. The HP and Ctrl samples are represented as green and red circles, respectively. (**B**) Heat map analysis of identified metabolites that were significantly altered between control (red) and HP (yellow) groups. The color range bar indicates downregulated metabolites as red and upregulated metabolites as green. (**C**) The volcano plot shows a significant change in the levels of several metabolites, of which red represents upregulated and blue describes downregulated plasma metabolites in HP and Ctrl groups (FDR *p*-value ≤ 0.05, fold change ≥ 1.5). The grey dots represent unsignificant metabolites.

Figure 3 illustrates the metabolite biomarkers that distinguish the HP and Ctrl samples. In Figure 3A shows an OPLS-DA model score plot. The ability of plasma metabolites to differentiate HP from Ctrl samples is demonstrated by the distinct separation of the HP group from the Ctrl group. The heat map analysis found metabolites showing notable variations between the Ctrl and HP groups (Figure 3B). The metabolites with a noteworthy difference between the HP and Ctrl groups are well-represented by the heat map. Thus, they might be considered possible metabolite biomarkers in identifying HP. A moderate *t*-test (*p*-value < 0.05) and fold change (FC cutoff of 1.5) were used to analyze the volcano plot between the HP and Ctrl groups. The results showed that from 157 dysregulated metabolites, 127 (red) and 30 (blue) metabolites were upregulated and downregulated in the HP and Ctrl group, respectively. A total of 15 metabolites were identified as human endogenous metabolites (Figure 3C).

### 3.5. Metabolomics Profiling between EC and HP

**Figure 4:**
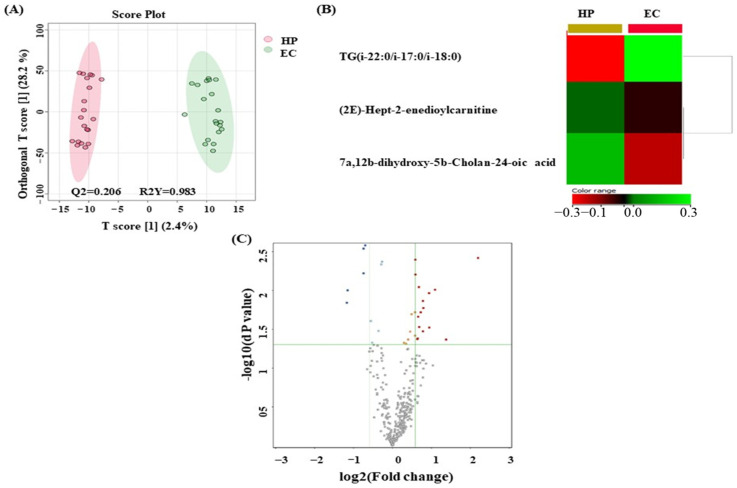
Metabolomics profiling between endometrial cancer (EC) and hyperplasia (HP) patients. (**A**) Orthogonal partial least squares discriminant analysis (OPLS-DA) score plot showing evident separation between two EC and HP patient groups. The robustness of the created models was evaluated by the fitness of the model (R2Y = 0.983) and predictive ability (Q2 = 0.206) values. The EC and HP samples are represented as green and red circles, respectively. (**B**) Hierarchal clustering and heat map analysis of identified metabolites that were significantly altered between EC (red) and HP (yellow) groups. The color range bar indicates downregulated metabolites as red and upregulated metabolites as green. (**C**) The volcano plot shows a significant change in the levels of several metabolites, of which red represents upregulated and blue describes downregulated plasma metabolites in EC and HP groups (FDR *p*-value ≤ 0.05, fold change ≥ 1.5). The grey dots represent unsignificant metabolites.

The possible biomarkers that changed between EC and HP are displayed in Figure 4. OPLS-DA, a multivariate supervised approach, is shown in Figure 4A. A few metabolites in the EC group are distinct from those in HP, suggesting that plasma metabolomics may be an effective method for distinguishing EC from HP. Between EC and HP, a cross-validated R2Y and Q2 coefficient was noted. Metabolites that were significantly different between the HP (yellow) and EC (red) groups were found using the heat map analysis (Figure 4B). The metabolites that significantly differ between the EC and HP groups are displayed on the heat map. As a result, these metabolites show promise as potential biomarkers for EC detection. A volcano plot analysis was conducted to compare the EC and HP groups using a fold change criterion of 1.5 and a moderate *t*-test (*p*-value < 0.05). The results showed that from a total of 21 dysregulated metabolites, 16 (red) and 5 (blue) metabolites were upregulated and downregulated in the EC and HP groups, respectively. A total of three metabolites were identified as human endogenous metabolites (Figure 4C).

### 3.6. Evaluation of Metabolite Biomarkers between EC and Ctrl Groups and Network Pathway

**Figure 5:**
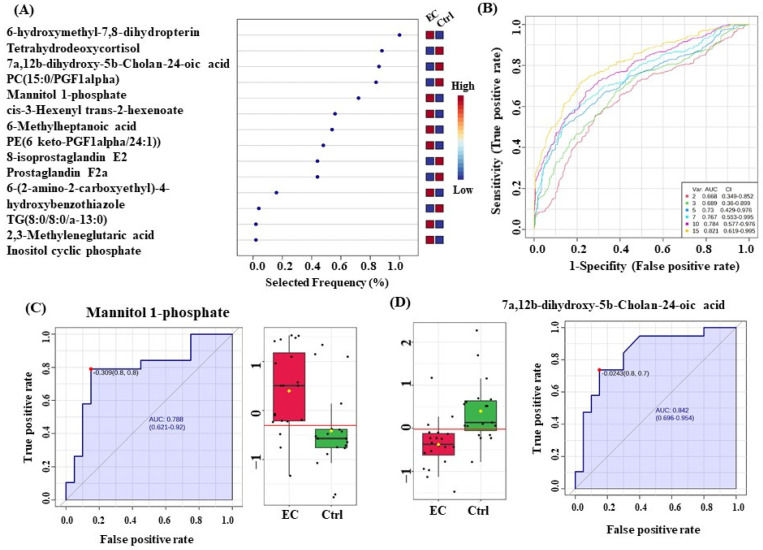
Biomarker evaluation in Endometrial Cancer (EC) and Controls (Ctrl). **(A)** Frequency plot for the top 15 metabolites. (**B**) The Receiver Operating Characteristics (ROC) curve was generated by the OPLS-DA model, with Area Under the Curve (AUC) values calculated from the combination of 2, 3, 5, 7, 10, and 15 metabolites. (**C**,**D**) Two metabolites in EC with the largest AUC (FDR *p* ≤ 0.05 and fold change ≥ 1.5), where red represents EC and green represents control.

**Figure 6:**
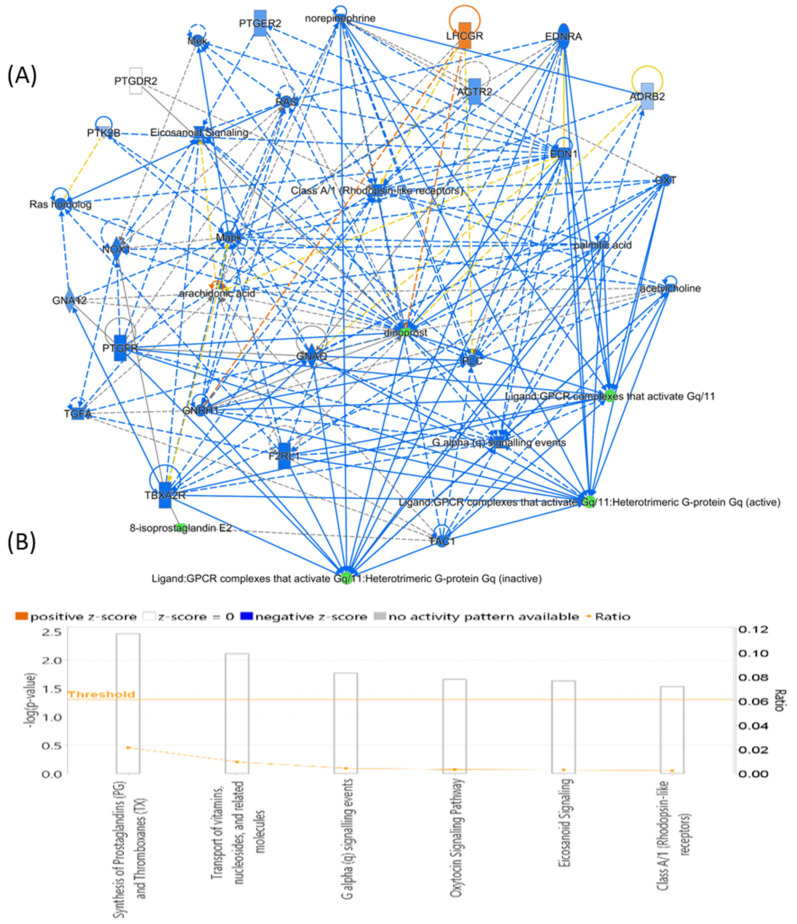
Schematic representation of the highest-scoring network pathways depicting the involvement of the differentially regulated metabolites (**A**) between patients with Endometrial Cancer and Controls. Nodes colored blue represent downregulation and orange represents upregulation. (**B**) The 6 top canonical pathways ranked by the *p*-values obtained by the IPA. The interaction networks were generated through IPA (QIAGEN Inc., Hilden, Germany).

A multivariate exploratory ROC analysis was conducted using OPLS-DA as a feature-ranking and classification approach based on the identified common and significantly dysregulated metabolites between the EC and Ctrl groups. For the top 15 variations (metabolites) (Figure 5A), the exploratory ROC curve’s area under the curve (AUC) was 0.821 (Figure 5B). For two metabolites’ AUCs from the top 15 variations, Figure 5C,D show box and whisker plots where red represents EC and green indicates Ctrl (FDR *p* ≤ 0.05 and fold change ≥ 1.5). Additionally, the IPA identified nucleic acid metabolism, small-molecule biochemistry, and carbohydrate metabolism as the network pathways affected with the highest score between EC and control groups (score of 6) (Figure 6A,B). The top 5 canonical pathways included histidine degradation III (1.36 × 10^3^ 5.6%), histidine catabolism (1.96 × 10^3^ 3.8%), histidine degradation VI (2.49 × 10^3^ 3.0%), synthesis of prostaglandins (PGs) and thromboxanes (TXs) (3.47 × 10^3^ 2.2% 1/46), and transport of vitamins, nucleosides, and related molecules (7.68 × 10^3^ 1.0%).

### 3.7. Evaluation of Metabolite Biomarkers between HP and Ctrl Groups and Network Pathway

**Figure 7:**
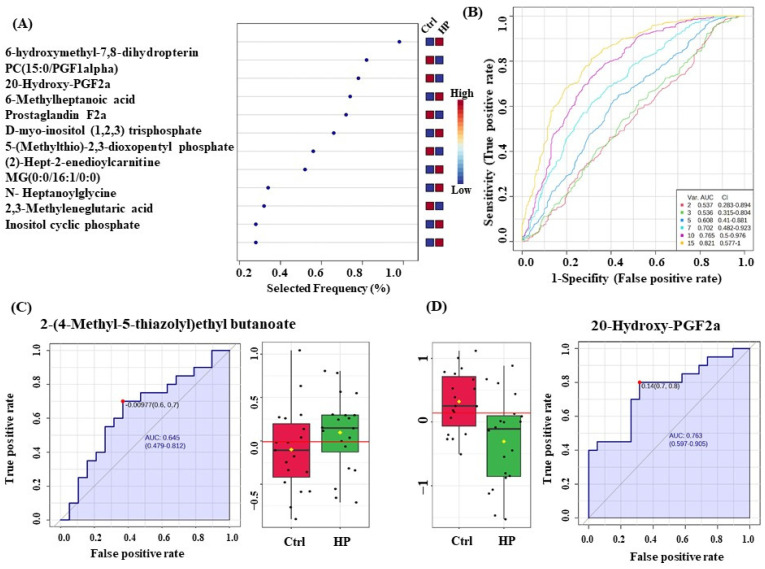
Biomarker evaluation in Hyperplasia (HP) and Controls (Ctrl). (**A**) The Frequency plot for the top 15 metabolites. (**B**) The Receiver Operating Characteristics (ROC) curve was generated by the OPLS-DA model, with Area Under the Curve (AUC) values calculated from the combination of 2, 3, 5, 7, 10, and 15 metabolites. (**C**,**D**) Two metabolites in EC with the largest AUC (FDR *p* ≤ 0.05 and fold change ≥ 1.5), where red represents control and green represents HP.

**Figure 8:**
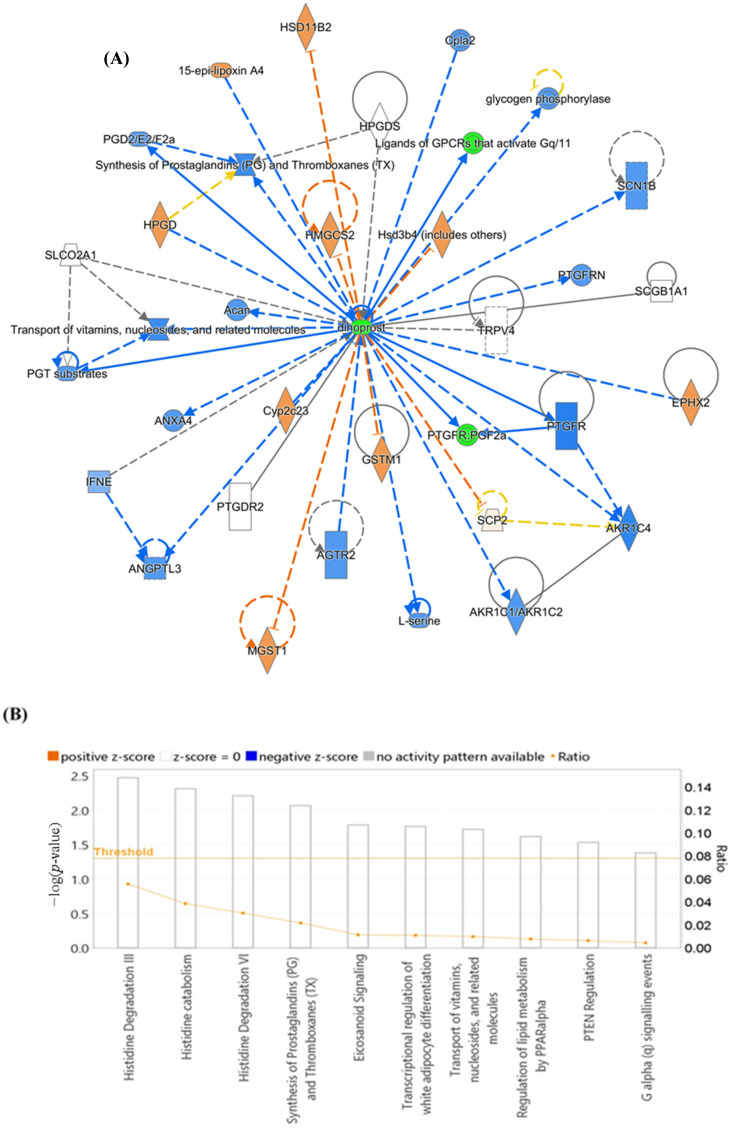
Schematic representation of the highest-scoring network pathways depicting the involvement of the differentially regulated metabolites. Nodes colored blue represent downregulation and orange represents upregulation. *(***A**) In patients with Hyperplasia and Controls, (**B**) 9 top canonical pathways ranked by the *p*-values obtained by the IPA. The interaction networks were generated through IPA (QIAGEN Inc.).

OPLS-DA was used as a classification and feature-ranking approach for a multivariate exploratory ROC analysis based on common and significantly dysregulated metabolites found between the HP and Ctrl groups (Figure 7A). Figure 7B shows that the AUC of the exploratory ROC curve for the top 15 variations (metabolites) was 0.821. For two metabolites’ AUCs from the top 15 variations, box and whisker plots (Figure 7C,D) show green denoting HP and red denoting Ctrl, with FDR *p* ≤ 0.05 and fold change ≥ 1.5. The pathway analysis showed that metabolites within this group related to changes in lipid metabolism, molecular transport, and small-molecule biochemistry (score of 4) (Figure 8A,B). The top 5 canonical pathways included synthesis of prostaglandins (PGs) and thromboxanes (TXs) (3.47 × 10^−3^ 2.2%), transport of vitamins, nucleosides, and related molecules (7.68 × 10^−3^ 1.0%), G alpha (q) signaling events (1.69 × 10^−2^ 0.4%), oxytocin signaling pathway (2.18 × 10^−2^ 0.3%), and eicosanoid signaling (2.32 × 10^−2^ 0.3%).

**Figure 9:**
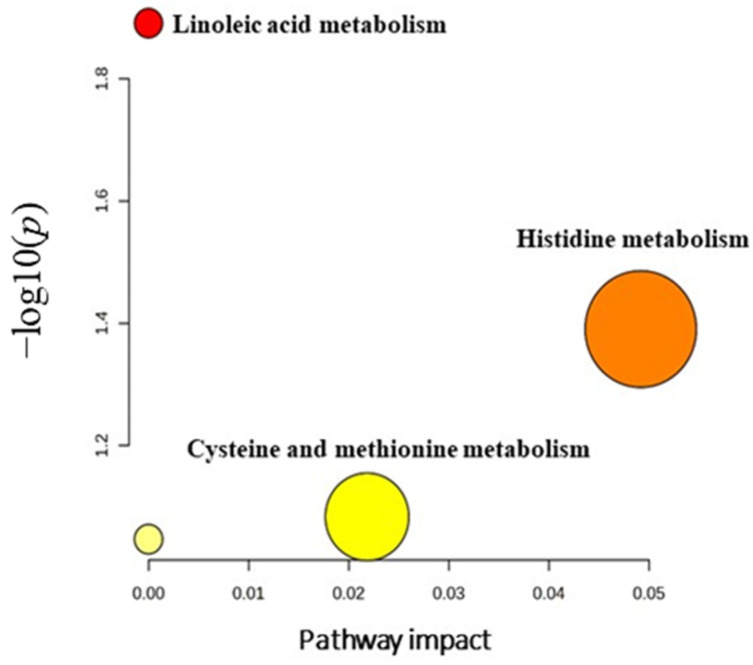
Pathway analysis of significantly dysregulated metabolites in EC patients compared to hyperplasia and healthy controls. Differing colors (varying from yellow to red) mean the metabolites have different significance levels (*p*-value).

Furthermore, metabolic pathway analysis revealed that the most relevant metabolic pathways related to the dysregulation of the identified 33 metabolites included histidine metabolism, linoleic acid metabolism, and cysteine and methionine metabolism (Figure 9).

## 4. Discussion

Cancer cells are fast-growing cells with a high demand for energy to maintain their survival and growth. In this regard, several metabolic alterations have been documented in many cancers supporting cancer cells’ ability to proliferate, mutate, metastasize, and defy treatment. One of the reported characteristics of cancer is an altered energy metabolism [25] including the mitochondrial oxidative phosphorylation pathway that is either defective or insufficient in cancer cells. This forces cancer cells to create the energy they require using alternate pathways, including aerobic glycolysis [26], lactate utilization [27], folate metabolism [28], or complete glutaminolysis through the TCA cycle under oxidative conditions [29]. In our present study, we identified alterations in the levels of these metabolites in the metabolic profiling of women with EC and hyperplasia compared to controls.

The levels of N-heptanoyl glycine were found to be significantly downregulated in hyperplasia in comparison with controls. N-heptanoyl glycine is an acylglycine, a minor metabolite of dietary fatty acids, accumulated due to defective lipolysis [30]. Therefore, the downregulation of these acylglycines can indicate fatty acid oxidation disorders in hyperplasia. This, along with the drop in 5-(Methylthio)-2,3-isopentyl phosphate levels and methionine, points to a multiple-step interruption in the methionine salvage process. The methionine consumption by cancer cells also leads to defects in the methionine cycle in T cells, affecting the adenosine-monophosphate-activated protein kinase (AMPK) activity. Defects in the activity of AMPK ultimately lead to upregulation of several immune-suppressive proteins like programmed cell death 1 (PD-1), which restricts immunological responses, upholds peripheral tolerance, and aids in tumor growth [31].

Among all amino acids circulating in human blood, glutamine is the most prevalent. Cancer cells exhibit markedly elevated glutamine uptake compared to their normal human tissue counterparts [32,33]. Cancer cells have also shown a proclivity for glutamate utilization. Research has demonstrated that glutamate plays a role in glutaminolysis and GSH synthesis within various cancer types [34]. Glutamine and glutamate metabolism are significantly upregulated in cancer cells, particularly breast cancer. In breast-invasive carcinoma, glutamine is pivotal in purine synthesis, providing the necessary building blocks for DNA and RNA production. This metabolic shift fuels the rapid proliferation and growth of cancer cells [34]. In addition, formimino-glutamic acid is significantly upregulated in hyperplasia compared to controls. Formimino-glutamic acid, also known as N-formimino-L-glutamate, belongs to the class of organic compounds known as glutamic acid and derivatives. The breakdown of histidine, an essential amino acid, involves a series of steps that rely on the presence of folic acid. Subsequently, formimino-glutamate is formed, which plays a crucial role in producing glutamate [35]. IPA identified a dysregulation in histidine metabolism in the top 3 canonical pathways between hyperplasia and controls. As these metabolites did not show any significant difference between control and EC subjects, N-heptanol glycine and -(Methylthio)-2,3-isopentyl phosphate and formimino-glutamic acid may be specific markers for hyperplasia conditions.

In EC, PE-NMe2(18:1/24:0) is significantly elevated compared to controls. PE-NMe2(18:1/24:0), also known as dimethyl phosphatidyl ethanolamine, is a type of phospholipid that belongs to the glycerol phospholipid class. It is a crucial intermediate in phosphatidylcholine (PC) biosynthesis, a major component of cell membranes. PC is vital in maintaining membrane structure, fluidity, and signaling functions. Dimethyl phosphatidyl ethanolamine interacts with signaling proteins, modulating their activity and influencing various cellular processes, including cell growth, differentiation, and apoptosis. It also regulates the trafficking of proteins and lipids within the cell, which is crucial for cell signaling, protein sorting, and organelle biogenesis [36,37]. Decreased levels of this phospholipid are observed in the brains of Alzheimer’s disease and Parkinson’s disease patients, suggesting its potential involvement in the pathogenesis of these neurodegenerative disorders [38,39]. Conversely, elevated PE-NMe2(18:1/24:0) levels are found in some types of cancer, such as esophageal tumors, implying its potential role in cancer development and progression [37].

8-iso prostaglandin E2 was significantly downregulated in EC compared to controls. 8-isoprostaglandin E2 is a prostaglandin-like compound produced from an intermediate molecule called 8-iso-PGH2. Isoprostanes, a family of prostaglandin-like compounds, are formed in the body through a free-radical-driven peroxidation process involving arachidonic acid, a fatty acid [40]. Isoprostanes, generated within the body through non-enzymatic free-radical-mediated lipid peroxidation, are indicative of oxidative stress and elevated isoprostane levels may exhibit antiangiogenic properties [41]. The isoprostane 8-iso-PGE2 blocked the migration and tube formation of endothelial cells induced by vascular endothelial growth factor (VEGF) both in vitro and in vivo [42]. They have been demonstrated to activate signaling pathways involving inositol phospholipids and protein kinase C, which are known to play a significant role in cancer development. Therefore, isoprostanes could represent a new category of naturally occurring tumor promoters. In experiments involving male B6C3F1 mice, the absence of any observable effect of 8-iso-PGE2 alone suggests that its combined effect with epidermal growth factor (EGF) on cell proliferation may be synergistic [43]. Similar to our findings with EC patients, a lower level of 8-iso-PGE2 was observed in colorectal cancer patients [44]. As the metabolites dimethyl phosphatidyl ethanolamine and 8-isoprostaglandin E2 did not show any significant difference between control and HP subjects, they may be specific markers for EC conditions. Network pathway analyses, using IPA, also demonstrated metabolite 8-iso-PGE2 to be connected with the other identified metabolites in the dataset, including PGF2 α, via the TBXA2R node.

Low levels of PGF2 and its metabolites were seen in HP and EC samples, and according to others, this is a poor prognostic indicator [45]. Cyclooxygenases (COXs) are enzymes that oxidize arachidonic acid (AA) to produce a family of lipids known as prostaglandins (PGs). While prostaglandin D2 (PGD2) is said to impede tumor advancement, prostaglandin F2 alpha (PGF2α) and prostaglandin E2 (PGE2) appear to promote tumor aggressiveness and progression [46]. The decrease in PGD2 prevents the growth of new blood vessels, while its decrease promotes the neovascularization required to advance tumors. Compared to healthy persons, melanoma tumor cells and other cancer types have been reported to have higher amounts of PGF2α, which promotes tumor development and decreases apoptosis in cancer cells [47]. This might be true at an early stage of cancer [48]. PGF2α was also identified as the central node in the IPA network pathway that influenced metabolic pathways in EC and HP. The decreased levels of PGF2α in the EC patients was found to increase the activity of arachidonic acid, luteinizing hormone, and choriogonadotropin receptor on the network map. Among the canonical pathways, prostaglandin and thromboxane synthesis and eicosanoid signaling were among the top 5 pathways identified by the software.

The overactivation of fatty acid oxidation in mitochondria in response to the elevated energy demand in cancer cells is linked to the decrease in acylcarnitine in HP and EC samples. Previous research shows that acetyl-CoA carboxylase 2 (ACC2) expression is lowered in cancer cells due to the deacetylation of histones to adapt to the acidic pH in the tumor microenvironment. This enzyme catalyzes the irreversible carboxylation of acetyl-CoA to produce malonyl-CoA that, in turn, inhibits the rate-limiting step in beta-oxidation of fatty acids. By controlling the enzyme carnitine acyltransferase, malonyl-CoA prevents fatty acids from binding to carnitine and prevents them from getting into the mitochondria, which is where fatty acid oxidation and breakdown take place. By decreasing the expression of ACC2, this feedback inhibition is stopped, and the fatty acid oxidation cycle could be irregularly activated in a known phenomenon called the “Corbet–Feron” effect [49,50]. This agrees with the Tuncer et al. study, where women with EC had far lower serum total L-carnitine levels than healthy individuals, and this difference was related to the disease’s stage. Therefore, the deficiency in L-carnitine could contribute to the etiology of endometrial cancer independent of obesity or an increase in body fat and could serve as an early diagnostic indicator for EC [51]. Our IPA also showed that the pathway most affected between hyperplasia and controls was the one involving lipid metabolism.

In addition, many gynecologic malignancies have a significant impact on lipid metabolism. According to a study, ovarian cancer patients’ plasma lipid profiles were lower than healthy individuals, consistent with our results [52]. Lipids are essential components of the cell’s plasma membrane, which preserves the enclosure’s integrity. They are also crucial signaling molecules and energy sources, and it is widely known that they are linked to cancer [53]. The observed reduced lipids might be due to their increased utilization by cancerous cells during proliferation. However, whether hypolipidemia at the time of diagnosis contributes to cancer’s development or its effect is still unresolved. Although study results had inconsistent findings, monitoring plasma lipid status may be a useful indicator of the early changes [54].

In this study, we observed that low prediction rate (Q2) prompts a comprehensive examination of several crucial aspects to elucidate the underlying factors contributing to this outcome. First and foremost, a rigorous evaluation of data quality was undertaken, ensuring the reliability of raw data while meticulously scrutinizing sample preparation, instrument calibration, and data acquisition. An in-depth analysis of the preprocessing steps was conducted, focusing on normalization, scaling, and transformation techniques to ensure their appropriateness for our dataset. An OPLS-DA selection was pivotal in our investigation, with multiple algorithms explored to identify the most fitting one for our data. Feature selection and dimensionality reduction techniques were also implemented to refine the variables used in this model. The cross-validation strategy was carefully revisited, optimizing the number of folds and randomization procedures. Considering the intrinsic biological variability in metabolomics studies, we acknowledge the presence of natural variations that may influence the predictability of our model. A validation step using an independent dataset was incorporated to bolster the robustness of our findings.

Furthermore, the limitations of the small sample size and potential external factors such as lifestyle and diet were acknowledged. Statistical significance was rigorously assessed, and sensitivity analyses were performed to discern the impact of various parameters on model performance. Considering these factors, we propose avenues for future research and experimentation to address the identified limitations and refine our model’s predictive capabilities.

## 5. Conclusions

EC is linked to severe metabolic dysfunction through altering ratios between glucose, glutamine, and fatty acid use to increase the total yield of cellular ATP. This continuous reprogramming gives cancer cells a selective advantage for growth. The changes in metabolites identified in our study indicate that endometrial cancer cells adopt alternative strategies to increase energy production to meet the energy demand, thereby supporting proliferation.

## Data Availability

All data generated or analyzed in the current study are included in this article.

## Supplementary Materials

The following supporting information can be downloaded at https://www.mdpi.com/article/10.3390/metabo14020109/s1, Supplementary data Table S1: Characteristics of study subjects; Supplementary data Table S2: Mass spectrometry list of significant differentially abundant metabolites and identified metabolites, with changes in abundance of significantly differentially abundant metabolites between cancer, hyperplasia, and control states in plasma samples.
