# Supplementary material for: Metabolomic Profiling of Blood Plasma in Females with Hyperplasia and Endometrial Cancer
Source: Metabolites. 2024 Feb 6;14(2):109. doi: 10.3390/metabo14020109 (PMC10890097; doi:10.3390/metabo14020109)
Supplement: Supplementary file 1 [file metabolites-14-00109-s001.zip › Supplemantary data Table S1.pdf]

Table S1: Characteristics of study subjects

Sample ID	Sample type	Age	BMI	Diagnosis	Stage/Grade
EC1	Endometrial Cancer	70	33.69	Endometrioid endometrial adenocarcinoma	1A/G2
EC2	Endometrial Cancer	60	48.50	Endometrioid endometrial adenocarcinoma	1A/G2
EC 3	Endometrial Cancer	60	48.00	Endometrioid endometrial adenocarcinoma	1A/G1
EC 4	Endometrial Cancer	45	29.90	Endometrioid endometrial adenocarcinoma	1B/G3
EC 5	Endometrial Cancer	50	32.00	Endometrioid endometrial adenocarcinoma	1B/G1
EC 6	Endometrial Cancer	68	27.89	Endometrioid endometrial adenocarcinoma	3C/G3
EC 7	Endometrial Cancer	50	31.00	Endometrioid endometrial adenocarcinoma	3C/G2
EC 8	Endometrial Cancer	72	25.36	Endometrioid endometrial adenocarcinoma	3A/G2
EC 9	Endometrial Cancer	59	45.61	Endometrioid endometrial adenocarcinoma	1A/G3
EC 10	Endometrial Cancer	54	30.00	Endometrioid endometrial adenocarcinoma	1A/G2
EC 11	Endometrial Cancer	58	44.23	Endometrioid endometrial adenocarcinoma	1A/G1
EC 12	Endometrial Cancer	52	35.02	Endometrioid endometrial adenocarcinoma	1A/G1
EC 13	Endometrial Cancer	55	44.06	Endometrioid endometrial adenocarcinoma	1A/G1
EC 14	Endometrial Cancer	62	29.36	Endometrioid endometrial adenocarcinoma	1B/G1
EC 15	Endometrial Cancer	67	33.56	Endometrioid endometrial adenocarcinoma	1A/G2
EC 16	Endometrial Cancer	63	37.25	Endometrioid endometrial adenocarcinoma	1A/G3
EC 17	Endometrial Cancer	59	38.00	Endometrioid endometrial adenocarcinoma	1A/G2
EC 18	Endometrial Cancer	58	34.01	Endometrioid endometrial adenocarcinoma	1A/G3
EC 19	Endometrial Cancer	62	46.28	Endometrioid endometrial adenocarcinoma	1A/G2
EC 20	Endometrial Cancer	66	35.98	Endometrioid endometrial adenocarcinoma	1A/G2
HP 1	Hyperplasia	49	26.03	hyperplastic endometrium without atypia	
HP 2	Hyperplasia	49	32.00	hyperplastic endometrium with atypia	
HP 3	Hyperplasia	48	33.30	hyperplastic endometrium with atypia	
HP 4	Hyperplasia	48	34.20	hyperplastic endometrium with atypia	
HP 5	Hyperplasia	48	34.00	hyperplastic endometrium without atypia	
HP 6	Hyperplasia	58	39.90	hyperplastic endometrium with atypia	
HP 7	Hyperplasia	65	38.20	hyperplastic endometrium with atypia	
HP 8	Hyperplasia	65	37.90	hyperplastic endometrium with atypia	
HP 9	Hyperplasia	58	37.93	hyperplastic endometrium with atypia	
HP 10	Hyperplasia	61	48.13	hyperplastic endometrium with atypia	
HP 11	Hyperplasia	50	36.03	hyperplastic endometrium with atypia	
HP 12	Hyperplasia	59	38.01	hyperplastic endometrium with atypia	
HP 13	Hyperplasia	60	37.56	hyperplastic endometrium with atypia	
HP 14	Hyperplasia	59	32.89	hyperplastic endometrium with atypia	
HP 15	Hyperplasia	61	33.75	hyperplastic endometrium with atypia	
HP 16	Hyperplasia	48	39.00	hyperplastic endometrium with atypia	
HP 17	Hyperplasia	55	37.25	hyperplastic endometrium with atypia	
HP 18	Hyperplasia	54	40.00	hyperplastic endometrium with atypia	
HP 19	Hyperplasia	58	42.02	hyperplastic endometrium with atypia	

HP 20	Hyperplasia	59	44.20	hyperplastic endometrium with atypia
Ctrl 1	Control	48	28.21	Healthy
Ctrl 2	Control	43	39.89	Healthy
Ctrl 3	Control	63	41.62	Healthy
Ctrl 4	Control	44	46.93	Healthy
Ctrl 5	Control	65	30.02	Healthy
Ctrl 6	Control	52	24.90	Healthy
Ctrl 7	Control	52	23.70	Healthy
Ctrl 8	Control	52	23.30	Healthy
Ctrl 9	Control	43	39.90	Healthy
Ctrl 10	Control	43	39.80	Healthy
Ctrl 11	Control	40	39.02	Healthy
Ctrl 12	Control	43	29.20	Healthy
Ctrl 13	Control	47	33.02	Healthy
Ctrl 14	Control	52	35.23	Healthy
Ctrl 15	Control	58	28.00	Healthy
Ctrl 16	Control	62	37.89	Healthy
Ctrl 17	Control	70	35.00	Healthy
Ctrl 18	Control	71	39.00	Healthy
Ctrl 19	Control	69	35.00	Healthy
